# Enzyme-triggered compound release using functionalized antimicrobial peptide derivatives†
†Electronic supplementary information (ESI) available. See DOI: 10.1039/c6sc04435b



**DOI:** 10.1039/c6sc04435b

**Published:** 2017-02-20

**Authors:** Shin Mizukami, Masayoshi Kashibe, Kengo Matsumoto, Yuichiro Hori, Kazuya Kikuchi

**Affiliations:** a Institute of Multidisciplinary Research for Advanced Materials , Tohoku University , 2-1-1C Katahira, Aoba-ku , Sendai , Miyagi 980-8577 , Japan . Email: s-mizu@tagen.tohoku.ac.jp; b Division of Advanced Science and Biotechnology , Graduate School of Engineering , Osaka University , 2-1 Yamadaoka, Suita , Osaka 565-0871 , Japan . Email: kkikuchi@mls.eng.osaka-u.ac.jp; c Immunology Frontier Research Center , Osaka University , 2-1 Yamadaoka, Suita , Osaka 565-0871 , Japan

## Abstract

Controlled release is one of the key technologies for medical innovation, and many stimulus-responsive nanocarriers have been developed to utilize this technology. Enzyme activity is one of the most useful stimuli, because many enzymes are specifically activated in diseased tissues. However, controlled release stimulated by enzyme activity has not been frequently reported. One of the reasons for this is the lack of versatility of carriers. Most of the reported stimulus-responsive systems involve a sophisticated design and a complicated process for the synthesis of stimulus-responsive nanocarrier components. The purpose of this study was to develop versatile controlled release systems triggered by various stimuli, including enzyme activity, without modifying the nanocarrier components. We developed two controlled release systems, both of which comprised a liposome as the nanocarrier and a membrane-damaging peptide, temporin L (TL), and its derivatives as the release-controllers. One system utilized branched peptides for proteases, and the other utilized phosphopeptides for phosphatases. In our systems, the target enzymes converted the non-membrane-damaging TL derivatives into membrane-damaging peptides and released the liposome inclusion. We demonstrated the use of our antimicrobial peptide-based controlled release systems for different enzymes and showed the promise of this technology as a novel theranostic tool.

## Abstract 2

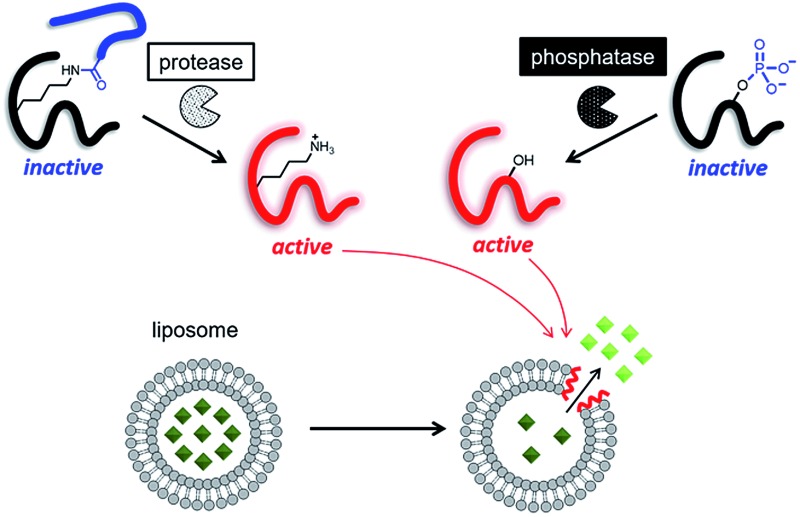
Two strategies have been proposed to develop enzyme-triggered compound release systems.

## Introduction

Since liposomes were first described in the literature,^1^ these nano- or meso-scale lipid-assembled composites have been at the center of research on drug delivery systems. Various technical advances in the field of liposome research, such as drug loading,^2^ extrusion for homogenous size,^3^ long circulation,^4^ and active targeting,^5^ have been achieved in just half a century.^6^ Controlled release and specific site-targeting are the focus of research for the clinical application of functional liposomes.^7^ The triggers for drug release are classified into two groups: the first includes remote triggers such as heat, ultrasound, and light, while the second includes local triggers such as pH change. pH-responsive liposomes^8^ have been investigated for application in drugs that act on regions with a mildly acidic pH, such as primary tumors or inflammation sites. Enzyme activity is another important local trigger, because the activities of specific enzymes are known to be increased in diseased tissues and cells. In addition, a new therapeutic approach, called directed enzyme prodrug therapy (DEPT), has recently started attracting attention.^9,10^ DEPT utilizes enzyme activity to locally activate prodrugs at the target tissues or cells. In DEPT, exogenous enzymes are delivered by various means such as antibodies, gene delivery, and viruses. Despite the increasing importance of such enzyme activity-based therapeutics, liposomes with enzyme reaction-based release triggers have not been well studied, and only a few enzymes such as phospholipases^11^ and matrix metalloproteinases^12^ have been reported as targets, and even these strategies are far from being ready for practical application. Most of the reported studies on stimulus-triggered release from liposomes are based on the conversion of lipids that cause the destabilization of membrane bilayers.^13^ Therefore, it is important to develop a novel strategy for enzyme-triggered release systems.

We have previously developed a UV light-induced compound release system that combined liposomes with a photocaged derivative of a frog-derived antimicrobial peptide, temporin L (TL).^14^ TL is a small (13-amino acid) peptide that was originally isolated from the skin of the European red frog *Rana temporaria*,^15^ and is involved in the innate immune system. Previous studies have shown that TL is highly toxic to both Gram-positive and -negative bacteria, fungi, and even cancer cells.^16^ The membrane-damaging property of TL is likely caused by the formation of pores in the bacterial lipid membranes. Similar to other antimicrobial peptides,^17^ TL has several cationic sites (*N*-terminus, Lys7, and Arg10) that are considered essential for its membrane-damaging property.^16^ We confirmed that the membrane-damaging activity of TL was controlled by the caging and uncaging of an ε-amino group with a photocleavable moiety, and speculated that this strategy might be universally applicable to enzyme-triggered compound release.

**Scheme 1:**
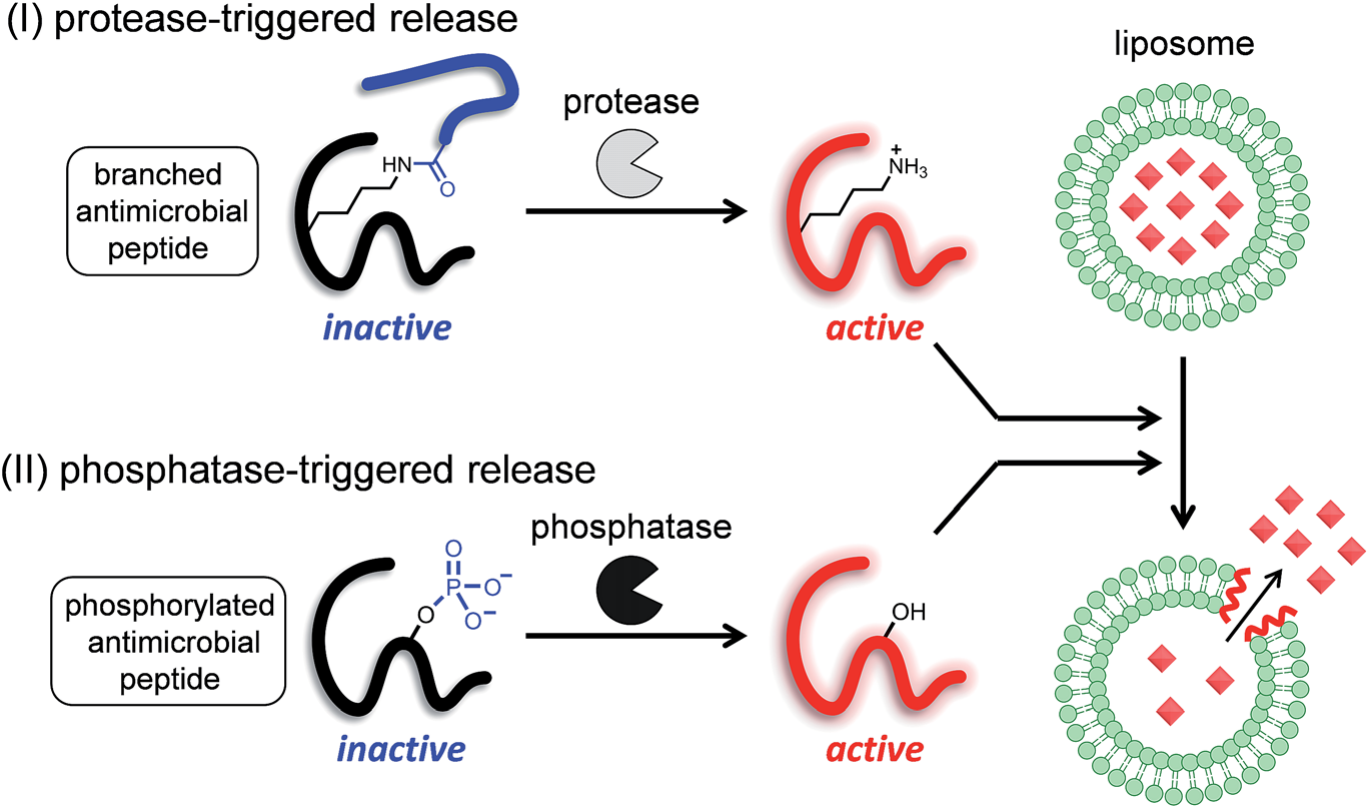
Schematic diagram of two different enzyme-triggered compound release systems: (I) a protease-triggered system using a branched antimicrobial peptide, and (II) a phosphatase-triggered system using a phosphorylated antimicrobial peptide.

Therefore, in this study, we proposed two strategies to develop enzyme-triggered compound release systems. One was an application of the previous uncaging system to enzymatically trigger. We demonstrated this concept by constructing a protease-triggered release system (system (I) in Scheme 1). The other was an extension of the concept of the previous system, where we regulated the membrane-damaging property using the net charge of the peptide and was demonstrated by developing a phosphatase-triggered release system (system II in Scheme 1). Here, we report the molecular designs, syntheses, and applications of these two enzyme-triggered compound release systems.

## Results

### I. Protease-triggered release system

**Chart 1:**
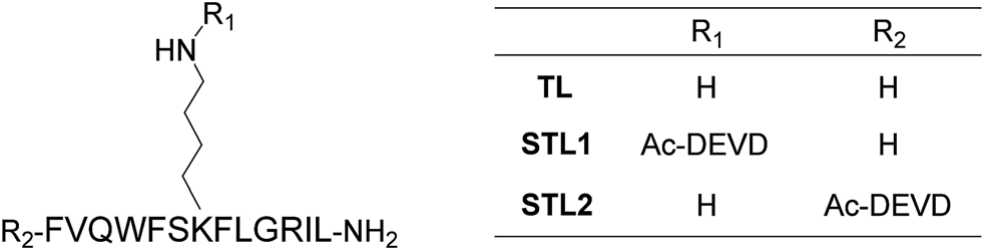
Structures of temporin L (TL) and its protease-responsive derivatives STL1 and STL2.

Among the many biologically relevant proteases, we chose an apoptosis-related protease, caspase-3, because we believed it to be suitable for a proof-of-principle study owing to its short and strict amino acid sequence for enzyme-recognition. Caspase-3 specifically hydrolyzes the C-terminal peptide bond of a tetrapeptide sequence, DEVD, and accepts any amino acid at the P1′ position.^18^ Therefore, we designed a branched peptide, STL1 (H-FVQWFSK(Ac-DEVD)FLGRIL-NH_2_), with a substrate sequence of caspase-3 (Chart 1). The branched peptide was synthesized using Fmoc solid-phase peptide synthesis. A non-branched isomer of STL1, STL2 (Ac-DEVD-FVQWFSKFLGRIL-NH_2_) was also synthesized (Chart 1). Both peptides were purified using reversed-phase high performance liquid chromatography (HPLC) and identified using mass spectrometry.

**Fig. 1:**
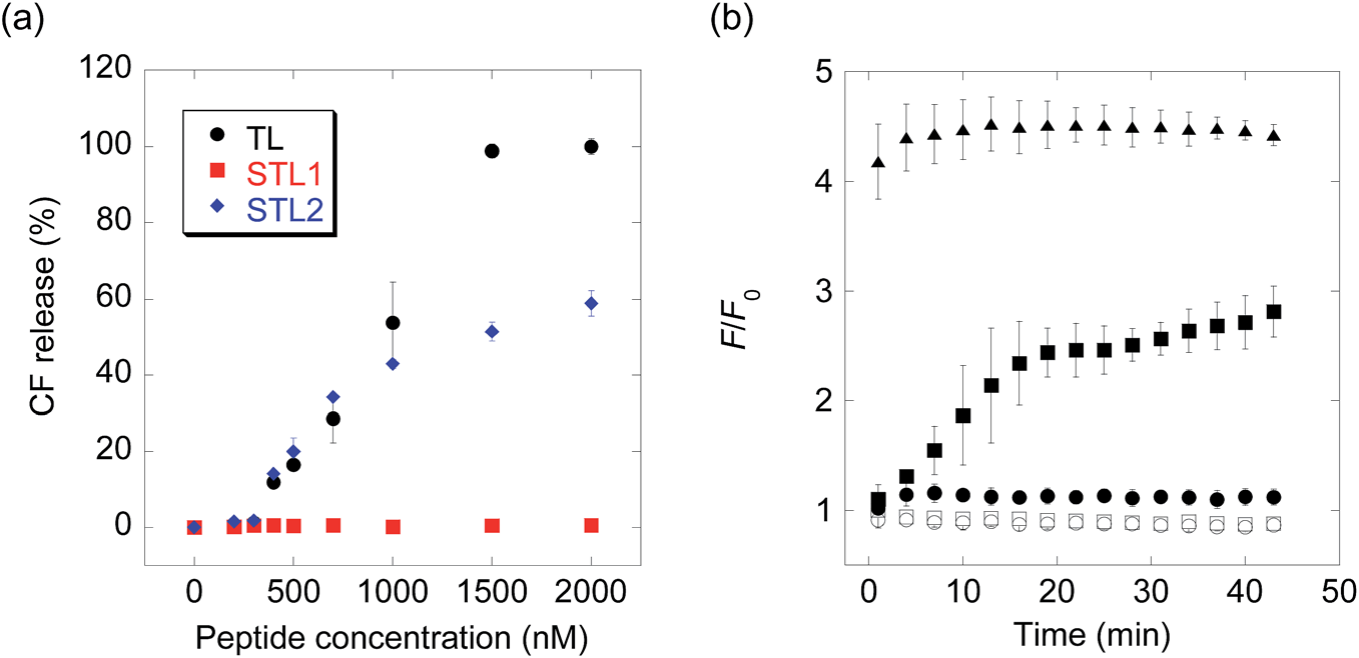
(a) Carboxyfluorescein (CF) release from large unilamellar vesicles (LUVs) upon addition of temporin L (TL), STL1, or STL2. (b) Real-time monitoring of caspase-3-triggered CF release (open squares: no probe, open circles: caspase-3, filled circles: STL1, filled squares: STL1 + caspase-3, filled triangles: TL). Concentration: [STL1] = 3 μM, [TL] = 3 μM, [caspase-3] = 1 U μL^–1^. Each value was plotted as the mean ± S.D. (*n* = 3).

The membrane-damaging activity of STL1 and STL2 was assayed using anionic-surface large unilamellar vesicles (LUVs) comprising DOPC (1,2-dioleoyl-*sn*-glycero-3-phosphocholine) and DOPG (1,2-dioleoyl-*sn*-glycero-3-phospho-*rac*-(1-glycerol)) (1 : 1) (Chart S1†) as the membrane components and carboxyfluorescein (CF) as the inclusion. As CF is partially quenched in liposomes due to its high concentration, the release of the inclusion by the membrane-damaging molecules can be detected by fluorescence enhancement caused by dilution. The membrane-damaging activity was assessed by quantifying the leakage fraction of CF 1 min after peptide addition, with a wide range of peptide concentrations (Fig. 1a). The branched derivative, STL1, almost lost its membrane-damaging ability at a concentration of ≤2 μM, whereas the linear derivative, STL2, retained its membrane-damaging property. The reason for this difference was investigated by comparing their circular dichroism (CD) spectra (Fig. S2†), which clearly indicated that TL and STL2 formed α-helix structures in the presence of the liposomes, while STL1 did not. These results are consistent with the hypothesis that the membrane-damaging ability of the various antimicrobial peptides depends on the formation of specific secondary structures on the lipid membrane.^19^

The above-mentioned results indicated that the conversion of STL1 into TL induced the release of the liposome inclusion. Therefore, we next investigated the ability of caspase-3 to convert STL1 into TL by cleavage of specific peptide bonds. After incubating STL1 with caspase-3 at 25 °C, HPLC was performed for which the results showed that caspase-3 recognized STL1 and that TL was formed as the product (Fig. S3†). Next, CF-loaded LUVs were incubated with 3 μM STL1 at 25 °C to achieve enzyme-triggered compound release by STL-1. The peptide concentration in this experiment was chosen based on a previous report, which showed that 5 μM TL had limited (about 20%) toxicity to lymphoma cells (U937) and that lower concentrations (2.5 μM) of TL showed little effect on the viability of other human cancer cell lines (Hut-78 and K-562).^16*c*^ We found that the fluorescence intensity of CF increased within a few minutes after the addition of caspase-3 (1 U μL^–1^), which indicated the release of CF from the liposomes. The fluorescence intensity reached a plateau within 15 min (Fig. 1b). Using quantitative HPLC analysis (Fig. S4†), we estimated that about 0.84 μM TL was generated after 15 min. However, no fluorescence enhancement was observed without caspase-3 or without STL1. We also assessed the membrane-damaging activity of small amounts of TL in the presence of STL1. The result suggested that TL and STL had no synergistic effect (Fig. S5†). Taken together, these results indicated that the rational design of a branched peptide with an antimicrobial peptide and an enzyme substrate could achieve protease-triggered compound release.

### II. Phosphatase-triggered release system

We also expanded the design principle of the enzyme-triggered release system for other types of enzymes. In this system, we planned to regulate peptide function by attaching an additional anionic group without protecting the ε-amino group of Lys7. We speculated that the membrane-damaging activity of the antimicrobial peptide derivatives could be recovered by enzymatic elimination of this additional anionic group. Phosphatases catalyze the dephosphorylation of phosphorylated substrates, which involves the elimination of anions, thus we expected phosphatases to be good candidates for establishing a proof of the concept. In addition, the activity of some phosphatases is known to be enhanced in diseased tissues,^20^ and so, phosphatase-triggered compound release could have potential practical applications. To combine the antimicrobial peptide-based release system with the activity of phosphatases, we used the hypothesis that phosphorylation at the appropriate position in the peptide sequence of TL would suppress its membrane-damaging activity (system (II) in Scheme 1). This hypothesis was based on intramolecular electrostatic interactions with cationic residues, which is essential for the membrane-damaging activity, and the electrostatic repulsion of anionic lipid membranes.

**Fig. 2:**
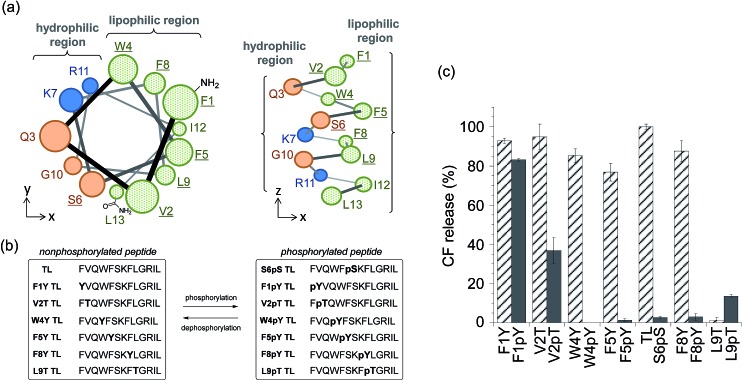
(a) Plausible α-helix structure of temporin L (TL) (left: top view, right: side view). Replaced amino acids are underlined. (b) Amino acid sequences of the synthesized TL derivatives. (c) Assays for membrane-damaging activity at a peptide concentration of 3 μM. Each bar refers to the normalized membrane-damaging activity of each non-phosphorylated (stripe) or phosphorylated (solid) TL derivative. Each value was plotted as the mean ± S.D. (*n* = 3).

To select the residue to be phosphorylated, we surveyed the plausible α-helix structure of the active TL, which was indicated in the analysis of the CD spectra (Fig. S2†). As shown in Fig. 2a, the α-helix structure of TL had an amphiphilic conformation with a hydrophilic region containing cationic residues and a lipophilic region consisting of aromatic and aliphatic residues. We speculated that the introduction of an anionic phosphate group into the lipophilic region would affect the membrane-damaging property of TL, and so, we selected Phe1, Val2, Trp4, Phe5, Phe8, and Leu9 as the residues to be phosphorylated. As these residues had no phosphorylation sites, we designed single-amino acid mutants of TL by replacing the aromatic or aliphatic amino acids with Tyr or Thr, respectively, in order to construct the phosphorylated TL derivatives. Six non-phosphorylated TL derivatives (F1Y, V2T, W4Y, F5Y, F8Y, and L9T TLs) and the corresponding phosphorylated TL derivatives (F1pY, V2pT, W4pY, F5pY, F8pY, and L9pT TLs) (Fig. 2b) were then synthesized using Fmoc solid-phase chemistry. S6pS TL was also synthesized as it was the sole phosphorylated derivative of native TL. All the peptides were purified using reversed-phase HPLC and identified using mass spectrometry.

The membrane-damaging properties of the phosphorylated and non-phosphorylated TL derivatives, as well as those of the protease-responsive TL derivatives, were investigated. As shown in Fig. 2c, the non-phosphorylated single-point mutant derivatives of TL, except L9T TL, mostly retained their membrane-damaging activities. However, the phosphorylation of the TL derivatives greatly affected their membrane-damaging properties. Four phosphopeptides (W4pY, F5pY, S6pS, and F8pY TLs) almost lost their membrane-damaging activities under experimental conditions. As their dephosphorylated counterparts (W4Y, F5Y, wild-type, and F8Y TLs) had sufficient activity, these compounds might be useful for phosphatase-triggered compound release from liposomes. The CD spectra were also assessed to verify the correlation between membrane-damaging activity and secondary structure (Fig. S6 and Table S1†); the α-helix structure was observed in most of the peptides that showed membrane-damaging activity. However, the CD spectra of some phosphopeptides such as S6pS and F8pY, which lost their membrane-damaging activities, also showed α-helix structures.

**Fig. 3:**
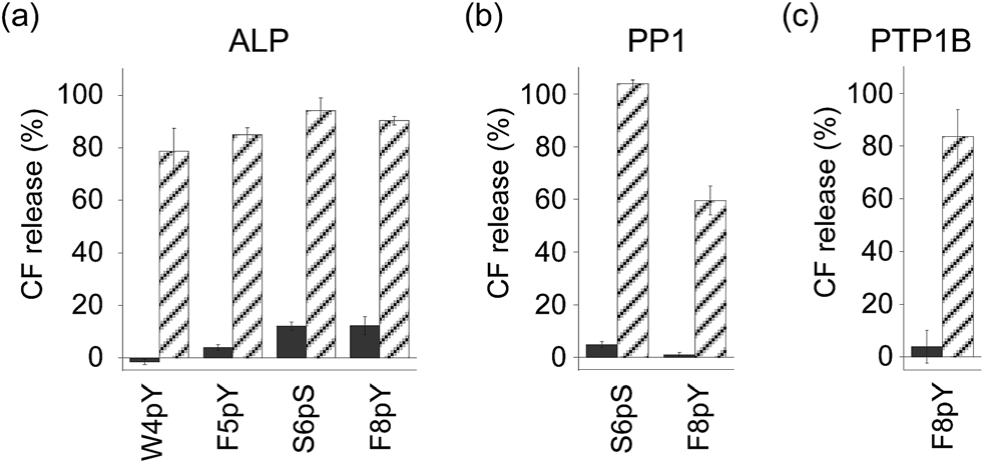
Compound release assays of phosphorylated TL derivatives with (stripe) and without (solid) phosphatases ((a) ALP, (b) PP1, and (c) PTP1B). [Peptide] = 3 μM, [ALP] = 0.6 U mL^–1^, [PP1] = 0.3 U mL^–1^, [PTP1B] = 0.12 μg (which corresponds to 7.2–14.4 U) mL^–1^ at final concentration. The peptide was incubated for 3 h. The compound release values were estimated from the fluorescence intensity. Each value was plotted as the mean ± S.D. (*n* = 3).

In order to assess the ability of phosphatases to recognize phosphopeptides as substrates, the phosphopeptides (W4pY, F5pY, S6pS, and F8pY TLs) were assayed with three different types of phosphatases: calf intestine alkaline phosphatase (ALP),^21^ protein phosphatase 1 (PP1: serine/threonine phosphatase),^22^ and protein tyrosine phosphatase 1B (PTP1B).^23^ After incubating the phosphopeptides with these phosphatases, the reaction mixtures were analyzed by HPLC (Table S2†). ALP dephosphorylated all four peptides (Fig. S7†), while PP1 dephosphorylated S6pS TL and F8pY TL (Fig. S8†) and PTP1B dephosphorylated only F8pY TL (Fig. S9†). It was noted that a conversion rate of 15% from F8pY to F8Y by PP1 (Table S2†) induced 60% fluorescence recovery (Fig. 3b). However, it is possible that small amounts of F8Y could induce significant liposome destruction.

The phosphorylated TL derivatives were then used for phosphatase-triggered compound release. As expected, the inclusion compounds in the liposomes were efficiently released in the presence of the phosphopeptides (W4pY, F5pY, S6pS, and F8pY TLs) after the addition of ALP (Fig. 3a). The phosphatase-triggered inclusion release was monitored in real-time using a fluorometer (Fig. S10†). In addition, we observed that S6pS TL and F8pY TL released the liposome inclusion in response to PP1 (Fig. 3b), while F8pY TL released the inclusion upon PTP1B activity (Fig. 3c). These results suggested that the selectivity of the different phosphatases could be modulated by combining the amino acid sequences of the phosphatase substrates and antimicrobial peptides.

**Fig. 4:**
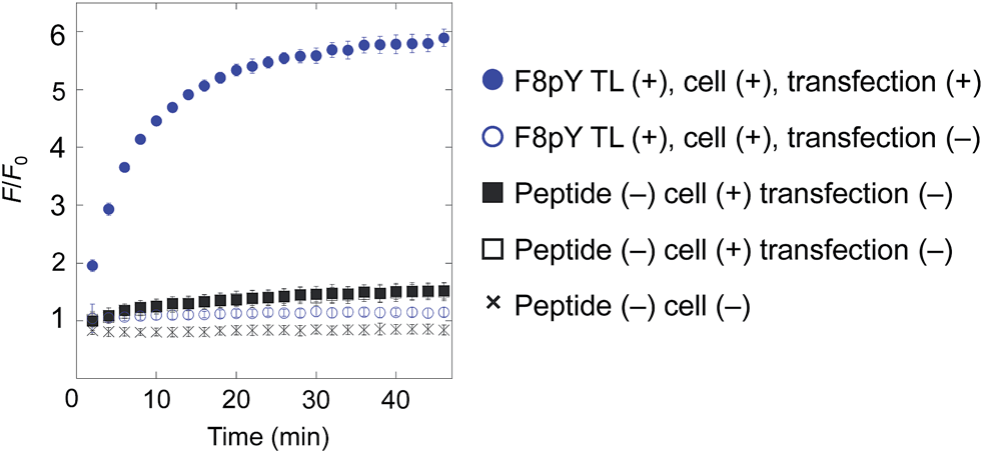
Compound release triggered by phosphatase secreted by living cells through dephosphorylation of F8pY TL. F8pY TL was preincubated in the culture dish involving transfected HEK 293T cells for 6 h before the addition of the liposome. Each value was plotted as the mean ± S.D. (*n* = 3).

Finally, for live cell applications, we verified the possibility of using this system for specific controlled release near target cells. Using GDEPT (gene-directed enzyme prodrug therapy), various enzymes can be targeted to specific cells or tissues by means of site-directed gene delivery.^9,10^ We applied this strategy to our controlled release system. In this study, target cells were transfected with a plasmid encoding secreted alkaline phosphatase (SEAP), which is widely used in reporter assays.^24^ HEK293T cells were cultured in a 96-well microculture plate, and after the expression of the gene, F8pY TL and liposomes containing CF were sequentially incubated. The compound release from the liposomes was then monitored by measuring the increase in fluorescence intensity using a microplate reader. As a result, distinctive CF release was observed from the wells with transfected cells (Fig. 4). In contrast, wells with non-transfected cells showed slower compound release, which was also the same for the wells without the F8pY TL peptide. The SEAP activity in the culture medium was separately assessed (Fig. S11†). These results clearly demonstrated that controlled compound release occurred due to the phosphatase secreted from the living target cells.

## Discussion

In this study, we described two novel enzyme-triggered compound release systems, designed using a combination of substrate-fused antimicrobial peptides and liposomes. Two different types of enzymes, a protease and phosphatase, were used as the target enzymes. For the protease-triggered system, we developed a functional branched peptide. Analysis of the CD spectra indicated that this branched peptide did not form an α-helix structure, which is thought to be a prerequisite for membrane-damaging ability. The key amino acid residue, Lys7, was protected by the protease substrate, and enzymatic cleavage resulted in the native form of TL, which released liposome inclusions. In principle, this strategy could also be applied to other proteases with low specificity of the P1′ amino acid.

To expand this protease-triggered release system into a more versatile system, we needed to utilize another strategy for regulating the membrane-damaging activities of antimicrobial peptides. To establish this second strategy, we focused on the local charge of the peptide, especially the difference in anionic charge before and after the enzyme reaction, because the electrostatic interaction between anionic lipids and cationic residues of antimicrobial peptides is considered important for membrane-damaging activity. We hypothesized that the incorporation of an anionic residue in TL derivatives would inhibit membrane damage. Phosphatases were chosen as suitable enzymes to validate this hypothesis. In this strategy, we selected amino acid residues, mainly from the lipophilic region of TL, and replaced one of them with a phosphorylated amino acid residue. Unlike the protease-responsive branched peptides, these peptide sequences were not targeted by specific phosphatases. Therefore, we surveyed four candidate phosphopeptides that showed noticeable reduction in membrane-damaging activity and recovery of this activity upon dephosphorylation, in response to three types of phosphatases, ALP, PP1, and PTP1B. Some phosphatases showed substrate specificity, and we demonstrated that this strategy could help in the development of compound release systems triggered by specific phosphatases.

We also investigated the correlation between membrane-damaging activity and secondary structures of the TL derivatives. The results of the analyses of CD spectra partly supported the notion that the presence of an α-helix structure is important for the membrane-damaging ability. However, in some modified TL derivatives, despite the α-helix structures in the antimicrobial peptides, their membrane-damaging abilities were found to be suppressed. This result indicated that the membrane-damaging ability could be regulated by disrupting any one of the steps involved in membrane destruction: (1) membrane binding, (2) α-helix formation, (3) membrane insertion, or (4) self-assembly.^17^ Therefore, we speculated that apart from the methods reported here, other methods for regulating the membrane-damaging activities of antimicrobial peptides could exist. These antimicrobial peptide-based strategies could help in the development of new controlled release systems triggered by various biological targets.

One of the problems that needs to be resolved in this system is the conjugation of the peptide and the liposome. The tethering of the peptide on the liposome surface induced the destabilization of the membrane. To overcome this challenge, it would be necessary to stabilize the liposome membrane without affecting its sensitivity to the membrane-damaging activity of the antimicrobial peptide or the optimal surface density of the peptide and the linker length. Effective polymer coating of the liposome surface would be necessary.^25^

## Conclusions

In conclusion, we have developed two different enzyme-activity-triggered compound release systems by combining modified antimicrobial peptides with surface-anionic liposomes. A protease and some phosphatases were chosen as the target enzymes. For the protease-triggered system, we designed a branched peptide that suppressed membrane-damaging activity by modifying the substrate peptide on the cationic Lys residue of TL. For the phosphatase-triggered system, we replaced a neutral amino acid with an anionic phosphorylated amino acid in the lipophilic region of TL. The phosphopeptides suppressed the membrane-damaging activity, and thus facilitated the controlled release, triggered by the phosphatase activity. To illustrate a potential application of this system, we demonstrated gene-directed enzyme-triggered compound release through a live cell experiment. In addition, these antimicrobial peptide-based controlled release systems could be applied for different enzymes by changing the trigger structure. Thus, this is a promising technology for specific drug targeting.

## Experimental section

### I. Synthesis of TL derivatives

TL and its derivatives were synthesized through solid-phase synthesis using a Rink Amide MBHA resin and Fmoc-protected amino acids. All the peptides were purified using reversed-phase HPLC and identified using mass spectrometry. The details of the syntheses are described in the ESI.†

### II. Assay for the membrane-damaging activity of enzyme-activatable TL derivatives

The buffer used in the assay for estimating the membrane-damaging activity was 10 mM HEPES buffer (pH 7.4) with 1 mM EDTA and 150 mM NaCl. Peptides and the liposome solution were mixed with the buffer in a microtube at RT. After 1 min, the fluorescence intensity at 25 °C was measured using a microplate reader (final peptide concentration = 0.03–10 μM, final lipid concentration = 2.5 μg mL^–1^). Each value was plotted as the mean ± S.D. (*n* = 3). The fluorescence intensity (*λ*_ex_ = 485 ± 7 nm, *λ*_em_ = 535 ± 12.5 nm) of the mixture was also measured using a microplate reader. The percentage of CF leakage was evaluated using the calculation, CF release (%) = (*F*_obs_ – *F*_0_)/(*F*_max_ – *F*_0_) × 100, where *F*_0_ and *F*_obs_ are the initial and measured fluorescence intensities of the samples, respectively, and *F*_max_ is the fluorescence intensity of the samples after the addition of TL (final concentration = 3 μM).

### III. Enzyme-triggered compound release

The buffers used for the enzyme reaction were: 10 mM HEPES buffer (pH 7.4) with 145 mM NaCl, 1 mM EDTA, and 10 mM DTT for caspase-3; 1 mM MgCl_2_ and 150 mM NaCl for ALP; 10 mM HEPES buffer (pH 7.4) with 2 mM DTT, 1 mM MnCl_2_, and 150 mM NaCl for PP1; and 10 mM HEPES buffer (pH 7.4) with 5 mM DTT, 1 mM EDTA, and 150 mM NaCl for PTP1B. Phosphorylated TL derivatives were reacted at a final concentration of 50 μM (for ALP) or 100 μM (for PTP1B and PP1) in aqueous buffers with 20 U mL^–1^ ALP, 10 U mL^–1^ PP1, or 4 μg mL^–1^ (which corresponds to 24–48 U mL^–1^) PTP1B. The final volume in each microtube was 200 μL. The peptides were incubated at 37 °C (for ALP and PTP1B) or at 30 °C (for PP1). All the peptide solutions were added to the liposome solution containing CF dispersed in the buffers (final concentrations of the peptide and the lipid were 3 μM and 2.5 μg mL^–1^, respectively). The fluorescence intensity (*λ*_ex_ = 485 ± 7 nm, *λ*_em_ = 535 ± 12.5 nm) was measured at 25 °C using a microplate reader, 1 min after the peptide addition. Each value was plotted as the mean ± S.D. (*n* = 3).

### IV. Gene-directed enzyme-triggered compound release

The medium used for the cell culture was DMEM and F8pYTL and the liposome solutions were prepared in 100 mM HEPES buffer (pH 7.4) with 1 mM MgCl_2_ and 105 mM NaCl. F8pY TL (final concentration = 5 μM) in HEPES buffer was added to HEK293T cells in DMEM that had been transfected with SEAP expression plasmids (phenol red (–), FBS (–), and antibiotics (–)). Six hours after the peptide addition, the liposome solution with CF (final concentration of the lipid = 2.5 μg mL^–1^) in HEPES buffer was also added. The fluorescence intensity (*λ*_ex_ = 485 ± 7 nm, *λ*_em_ = 535 ± 12.5 nm) was measured at 25 °C using a microplate reader, 2 min after liposome addition. Each value was plotted as the mean ± S.D. (*n* = 3).
