# Correction: Enzyme-triggered compound release using functionalized antimicrobial peptide derivatives

**DOI:** 10.1039/c7sc90017a

**Published:** 2017-03-13

**Authors:** Shin Mizukami, Masayoshi Kashibe, Kengo Matsumoto, Yuichiro Hori, Kazuya Kikuchi

**Affiliations:** a Institute of Multidisciplinary Research for Advanced Materials , Tohoku University , 2-1-1C Katahira, Aoba-ku , Sendai , Miyagi 980-8577 , Japan . Email: s-mizu@tagen.tohoku.ac.jp; b Division of Advanced Science and Biotechnology , Graduate School of Engineering , Osaka University , 2-1 Yamadaoka , Suita , Osaka 565-0871 , Japan . Email: kkikuchi@mls.eng.osaka-u.ac.jp; c Immunology Frontier Research Center , Osaka University , 2-1 Yamadaoka , Suita , Osaka 565-0871 , Japan

## Abstract

Correction for ‘Enzyme-triggered compound release using functionalized antimicrobial peptide derivatives’ by Shin Mizukami *et al.*, *Chem. Sci.*, 2017, DOI: ; 10.1039/c6sc04435b.



## 

Owing to an oversight the third line of the legend of Fig. 4 “■ Peptide (–), cell (+), transfection (–)” should be corrected as “■ Peptide (–), cell (+), transfection (+)” and the figure with corrected legend is shown below. This error does not change the conclusion of the paper.

**Fig. 1:**
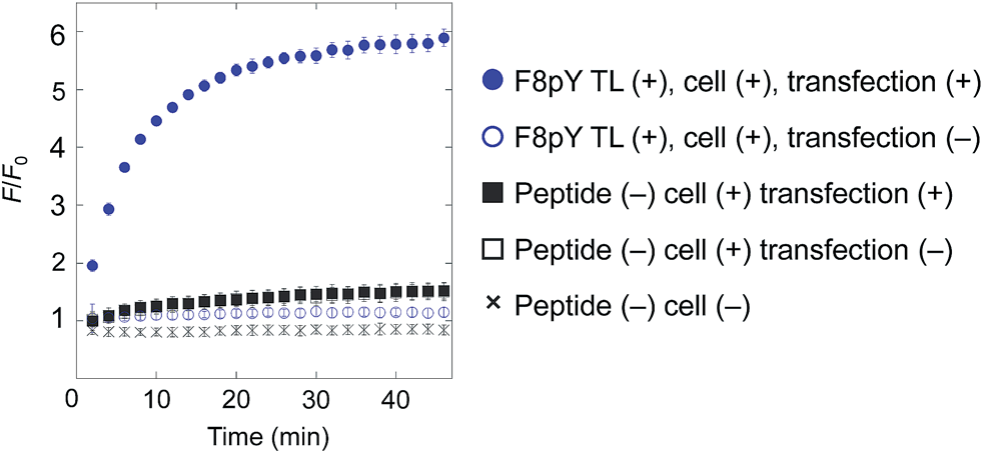
Compound release triggered by phosphatase secreted by living cells through dephosphorylation of F8pY TL. F8pY TL was preincubated in the culture dish involving transfected HEK 293T cells for 6 h before the addition of the liposome. Each value was plotted as the mean ± S.D. (*n* = 3).

The Royal Society of Chemistry apologises for these errors and any consequent inconvenience to authors and readers.

