# Supplementary material for: Enzyme-triggered compound release using functionalized antimicrobial peptide derivatives
Source: Chem Sci. 2017 Feb 20;8(4):3047–53. doi: 10.1039/c6sc04435b (PMC5380883; doi:10.1039/c6sc04435b)
Supplement: Supplementary file 1 [file SC-008-C6SC04435B-s001.pdf]

Electronic Supplementary Material (ESI) for Chemical Science.
This journal is © The Royal Society of Chemistry 2017

Electronic Supplementary Information (ESI)

Enzyme-triggered compound release by using functionalized derivatives using antimicrobial peptide

Shin Mizukami,[*,†] Masayoshi Kashibe,[‡] Kengo Matsumoto,[‡] Yuichiro Hori,[‡,§] and Kazuya Kikuchi[*,‡,§]

[†]Institute of Multidisciplinary Research for Advanced Materials, Tohoku University, 2-1-1 Katahira, Aoba-ku, Sendai, Miyagi 980-8577, Japan

[‡]Division of Advanced Science and Biotechnology, Graduate School of Engineering, Osaka University, 2-1 Yamadaoka, Suita, Osaka 565-0871, Japan

[§]Immunology Frontier Research Center, Osaka University, 2-1 Yamadaoka, Suita, Osaka 565-0871, Japan

*To whom correspondence should be addressed: s-mizu@tagen.tohoku.ac.jp, kkikuchi@mls.eng.osaka-u.ac.jp

CONTENTS:

1. Materials and instruments

2. Synthesis of peptides

3. Preparation of LUVs including CF

4. Measurement of CD spectra

5. Cell culture

6. Supporting Chart, Figures, and Tables

7. Supporting References

1. Materials and instruments

General chemicals were of the best grade available, supplied by Tokyo Chemical Industries, Wako Pure Chemical, Aldrich Chemical Co., Watanabe Chemical Industries, NOF Corporation, and Novabiochem. They were used without further purification. Sepharose^{TM} CL-4B resin was purchased from Amersham Biosciences. LF-1 LiposoFast apparatus and polycarbonate membrane filters (100 nm) were purchased from Avestin, Inc.

Mass spectra were taken on a Waters LCT-Premier XE for ESI, and a Bruker UltraFlexIII for MALDI-LIFT-TOF/TOF MS. Fluorescence spectra were measured using a Hitachi F4500 spectrometer. Slit width 5.0 nm for both excitation and emission. The photomultiplier voltage was 700 V. Fluorescence intensity was measured using a Perkin-Elmer 1420 ARVO MX-light microplate reader. High-performance liquid chromatography (HPLC) analyses were performed with an Inertsil ODS-3 column (4.6 mm × 250 mm (for analysis) or 10.0 mm × 250 mm (for purification), GL-Science, Inc.), and the absorbance at 215 nm was monitored. UV-vis absorption spectra were obtained with a Shimadzu UV-2450 spectrophotometer. CD spectra were obtained with a JASCO J-820 CD spectrometer. Dynamic light scattering (DLS) for size distribution was recorded on a Horiba nano Partica SZ-100.

Recombinat human caspase-3 was purchased from Enzo Life Sciences (Cat. BML-SE169). Calf intestinal alkaline phosphatase (ALP) (Cat. #: M0290S), and recombinant protein phosphatase 1 (PP1) (330 a.a. catalytic subunit of the ±-isoform of type 1 protein phosphatase from rabbit skeletal muscle, Cat. #: P0754S) were purchased from New England Biolabs. Recombinant PTP1B (321 a.a., Cat. #: PTB2001) was purchased from ATGen. Secreted alkaline phosphatase (SEAP) expression plasmid (pSF-CMV-SEAP) was purchased from Oxford Genetics. Culture plates were purchased from PerkinElmer, and Dulbecco's minimal essential medium (DMEM), fetal bovine serum (FBS), trypsin, and Lipofectamine 3000 were purchased from Invitrogen. The enzyme activities are defined as follows.[S1]

Caspase-3 : 1 U = 1 pmol min^{-1} using Ac-DEVD-pNA (200 μM) as substrate at 30°C

ALP : 1 U = 1 μmol min^{-1} using *p*-nitrophenyl phosphate (50 mM) as substrate at 37°C

PP1 : 1 U = 1 nmol min^{-1}using *p*-nitrophenyl phosphate (50 mM) as substrate at 37°C

PTP1B : 1 U = 1 nmol min^{-1} using *p*-nitrophenyl phosphate (10 mM) as substrate at 37°C

SEAP : 1 U = 1 μmol min^{-1} using *p*-nitrophenyl phosphate (11 mM) as substrate in 0.97M diethanolamine (pH 9.8) containing 0.5 mM Mg^{2+} at 37°C

2. Synthesis of peptides

General procedure All peptides were synthesized by solid-phase peptide synthesis using Rink Amide MBHA resin. According to the general Fmoc peptide chemistry, the Fmoc-protected amino acids were successively attached at RT. Then, the peptide was deprotected and cleaved from the resin using trifluoroacetic acid/water/triisopropylsilane/1,2-ethanedithiol (TFA/H_2O/TIS/EDT) = (94/2.5/1.0/2.5 (*v*/*v*/*v*/*v*)). Then, the solvent was removed under Ar atmosphere, and cold ether was added. The precipitate was collected using centrifugation. The final product was purified by reversed-phase HPLC to yield the pure peptide.

STL1 Fmoc-protected amino acids (Leu, Ile, Arg(Pbf), Gly, Leu, Phe, Lys(Mtt), Ser(*t*Bu), Phe, Trp(Boc), Gln(*t*Bu), Val, Phe) were successively attached. After the Fmoc group was removed by 20% piperidine in DMF, a DMF solution of Boc_2O and DIEA were added into resin and stirred at RT for 1 h. The resin was incubated with 1% TFA/DCM at RT for 1 h to remove the Mtt group. Then, Fmoc-protected amino acids (Asp(O*t*Bu), Val, Glu(O*t*Bu), Asp(O*t*Bu)) were successively attached. After the Fmoc group was removed by 20% piperidine in DMF, a solution of Ac_2O and DIEA were added into resin and stirred at RT for 1 h. MS (ESI-TOF): *m*/*z* calcd for $[M+2H]^{2+}$: 1070.06, found: 1070.48, $[M+3H]^{3+}$: 713.71, found: 714.01.

STL2 Fmoc-protected amino acids (Leu, Ile, Arg(Pbf), Gly, Leu, Phe, Lys(Mtt), Ser(*t*Bu), Phe, Trp(Boc), Gln(*t*Bu), Val, Phe, Asp(O*t*Bu), Val, Glu(O*t*Bu), Asp(O*t*Bu)) were successively attached. After the Fmoc group was removed by 20% piperidine in DMF, a solution of Ac_2O and DIEA were added into resin and stirred at RT for 1 h. MS (ESI-TOF): *m*/*z* calcd for $[M+2H]^{2+}$: 1070.57, found: 1070.52, $[M+3H]^{3+}$: 714.05, found: 714.04.

Temporin L (TL) Fmoc-protected amino acids (Leu, Ile, Arg(Pbf), Gly, Leu, Phe, Lys(Mtt), Ser(*t*Bu), Phe, Trp(Boc), Gln(Trt), Val, Phe) were successively attached. MS (MALDI-TOF): *m*/*z* calcd for $[M+H]^+$: 1639.947, found: 1640.082.

F1Y Temporin L (F1Y TL) Fmoc-protected amino acids (Leu, Ile, Arg(Pbf), Gly, Leu, Phe, Lys(Mtt), Ser(*t*Bu), Phe, Trp(Boc), Gln(Trt), Val, Tyr(*t*Bu)) were successively attached. MS (MALDI-TOF): *m*/*z* calcd for $[M+H]^+$ 1655.942, found 1655.778.

F1pY Temporin L (F1pY TL) Fmoc-protected amino acids (Leu, Ile, Arg(Pbf), Gly, Leu, Phe, Lys(Mtt), Ser(*t*Bu), Phe, Trp(Boc), Gln(Trt), Val, Tyr(PO(OBzl)OH)) were successively attached. MS (MALDI-TOF): *m*/*z* : calcd for $[M+H]^+$ 1735.908, found 1735.713.

V2T Temporin L (V2T TL) Fmoc-protected amino acids (Leu, Ile, Arg(Pbf), Gly, Leu, Phe, Lys(Mtt), Ser(*t*Bu), Phe, Trp(Boc), Gln(Trt), Thr(*t*Bu), Phe)) were successively attached. MS (MALDI-TOF): *m*/*z* calcd for $[M+H]^+$: 1641.926, found: 1641.314.

V2pT Temporin L (V2pT TL) Fmoc-protected amino acids (Leu, Ile, Arg(Pbf), Gly, Leu, Phe, Lys(Mtt), Ser(*t*Bu), Phe, Trp(Boc), Gln(Trt), Thr(PO(OBzl)OH), Phe) were successively attached. MS (MALDI-TOF): *m*/*z* calcd for $[M+H]^+$: 1721.893, found:1721.858.

W4Y Temporin L (W4Y TL) Fmoc-protected amino acids (Leu, Ile, Arg(Pbf), Gly, Leu, Phe, Lys(Mtt), Ser(*t*Bu), Phe, Tyr(*t*Bu), Gln(Trt), Val, Phe) were successively attached. MS (MALDI-TOF): *m*/*z* calcd for $[M+H]^+$: 1616.931, found: 1616.647.

W4pY Temporin L (W4pY TL) Fmoc-protected amino acids (Leu, Ile, Arg(Pbf), Gly, Leu, Phe, Lys(Mtt), Ser(*t*Bu), Phe, Tyr(PO(OBzl)OH), Gln(Trt), Val, Phe) were successively attached. MS (MALDI-TOF): *m*/*z* calcd for $[M+H]^+$: 1696.898, found: 1696.612.

F5Y Temporin L (F5Y TL) Fmoc-protected amino acids (Leu, Ile, Arg(Pbf), Gly, Leu, Phe, Lys(Mtt), Ser(*t*Bu), Tyr(*t*Bu), Trp(Boc), Gln(Trt), Val, Phe) were successively attached. MS (MALDI-TOF): *m*/*z* calcd for $[M+H]^+$ 1655.942, found 1656.074.

F5pY Temporin L (F5pY TL) Fmoc-protected amino acids (Leu, Ile, Arg(Pbf), Gly, Leu, Phe, Lys(Mtt), Ser(*t*Bu), Tyr(PO(OBzl)OH), Trp(Boc), Gln(Trt), Val, Phe) were successively attached. MS (MALDI-TOF): *m*/*z* calcd for $[M+H]^+$ 1735.908, found 1735.886.

S6pS Temporin L (S6pS TL) Fmoc-protected amino acids (Leu, Ile, Arg(Pbf), Gly, Leu, Phe, Lys(Mtt), Ser(PO(OBzl)OH), Phe, Trp(Boc), Gln(Trt), Val, Phe) were successively attached. MS (MALDI-TOF): *m*/*z* calcd for $[M+H]^+$ 1719.914, found 1719.739.

F8Y Temporin L (F8Y TL) Fmoc-protected amino acids (Leu, Ile, Arg(Pbf), Gly, Leu, Tyr(*t*Bu), Lys(Mtt), Ser(*t*Bu), Phe, Trp(Boc), Gln(Trt), Val, Phe) were successively attached. MS (MALDI-TOF): *m*/*z* calcd for $[M+H]^+$ 1655.942, found 1655.885.

F8pY Temporin L (F8pY TL) Fmoc-protected amino acids (Leu, Ile, Arg(Pbf), Gly, Leu, Phe, Lys(Mtt), Ser(*t*Bu), Tyr(PO(OBzl)OH), Trp(Boc), Gln(Trt), Val, Phe) were successively attached. MS (MALDI-TOF): *m*/*z* calcd for $[M+H]^+$ 1735.908, found 1735.789.

L9T Temporin L (L9T TL) Fmoc-protected amino acids (Leu, Ile, Arg(Pbf), Gly, Thr(*t*Bu), Phe, Lys(Mtt), Ser(*t*Bu), Phe, Trp(Boc), Gln(Trt), Val, Phe) were successively attached. MS (MALDI-TOF): *m*/*z* calcd for $[M+H]^+$: 1627.911, found: 1628.403.

L9pT Temporin L (L9pT TL) Fmoc-protected amino acids (Leu, Ile, Arg(Pbf), Gly, Thr(PO(OBzl)OH), Phe, Lys(Mtt), Ser(*t*Bu), Phe, Trp(Boc), Gln(Trt), Val, Phe) were successively attached. MS (MALDI-TOF): *m/z* calcd for $[M+H]^+$: 1707.877, found: 1707.759.

3. Preparation of LUVs including CF

DOPC (4 mg) and DOPG-Na (4 mg) (Chart S1) were dissolved in $CHCl_3$ (800 µL). The solvent was then removed under reduced pressure by rotary evaporation under Ar atmosphere, leaving a thin lipid film at the bottom of the flask, which was dried overnight *in vacuo*. For membrane-damaging activity assay, the lipid films were hydrated in 1 mL of aqueous solution including 50 mM 5,6-carboxyfluorescein (CF, dissolved with 2 eq. NaOH) and 100 mM NaCl (100 mM), and the resulting dispersion was vortexed extensively to form multilamellar vesicle (MLV). The suspension was extruded 31 cycles through polycarbonate membranes (pore diameter: 100 nm) using a LF-1 LiposoFast apparatus. The unencapsulated fluorophore was then separated from the fluorophore-loaded liposomes by size exclusion chromatography with a Sepharose™ CL-4B column pre-conditioned with 10 mM HEPES buffer (pH 7.4) containing 150 mM NaCl, 1 mM EDTA. The formation of LUVs was confirmed by DLS. The phospholipid content of liposome was determined by Stewart (ammonium ferrothiocyanate) assay as described elsewhere.[S2]

For LUVs used in CD analysis, the lipid films were hydrated in 1 mL of 10 mM sodium phosphate buffer (pH 7.4) solution including 150 mM NaCl. The dispersion was extensively vortexed, and the resulting MLV suspension was extruded 31 cycles through a membrane (pore diameter: 100 nm) using a LF-1 LiposoFast apparatus.

4. Measurement of CD spectra

The assay buffer for CD analysis was 10 mM sodium phosphate buffer (pH 7.4) containing 150 mM NaCl. Each peptide and LUVs were mixed in the assay buffer, and the CD spectra of peptides (50 µM) with LUVs ([lipid] = 1.0 mg mL-1) were measured at 25 °C (light path length: 1 mm, wavelength: 190–260 nm).

5. Cell culture

HEK293T cells were cultured with 10% FBS in DMEM in a humidified atmosphere with 5% CO_2 at 37 °C. Then the cells were trypsinized and resuspended in culture medium to a final concentration of 5×10^4 cells/ well. 100 µL cell suspension was added to a well of a black 96 well culture plate and incubated for 24 h. Then, the cells were transfected with a plasmid encoding SEAP by using Lipofectamine 3000. After 6 h, the culture medium was replaced with DMEM (phenol red (−), FBS (−), antibiotics (−)), and incubated for 36 h. The half volume of culture medium was sampled for quantification of SEAP activity,[S3] and the cells were used for enzyme-triggered compound release experiments.

6. Supporting Chart, Figures, and Tables

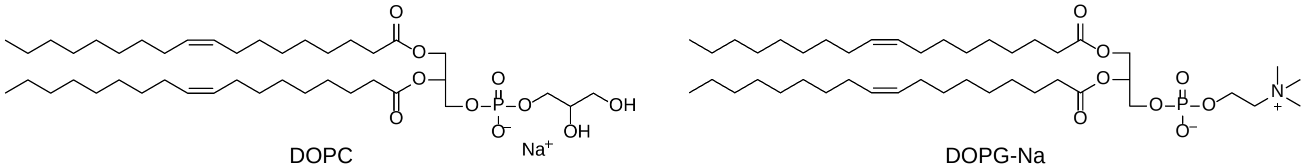

DOPC (1,2-dioleoyl-*sn*-glycero-3-phosphocholine)

DOPG-Na (1,2-dioleoyl-*sn*-glycero-3-phosphoglycerol sodium salt)

Chart S1. Structures of liposome membrane phospholipids

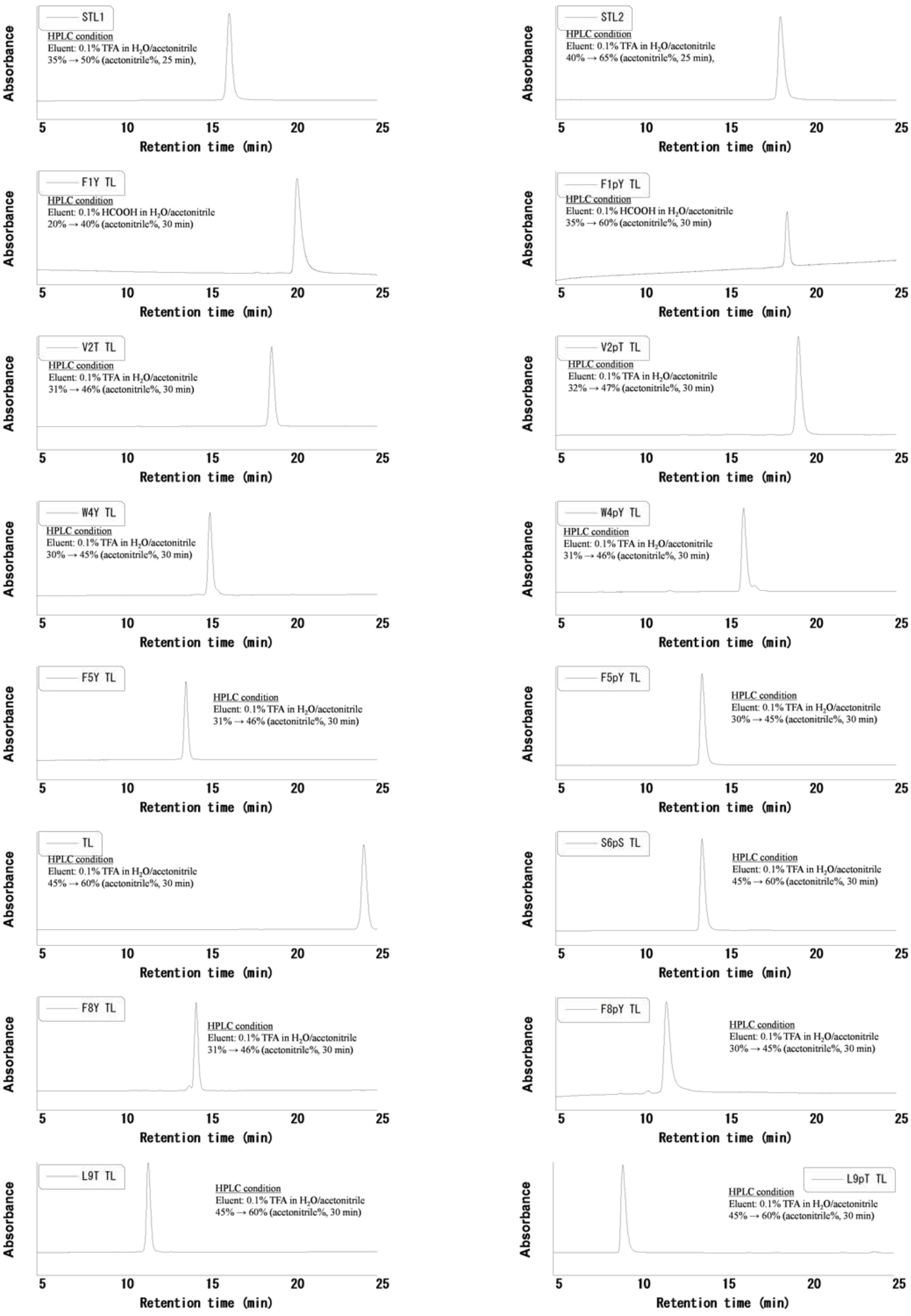

Figure S1. HPLC analyses of purified TL derivatives.

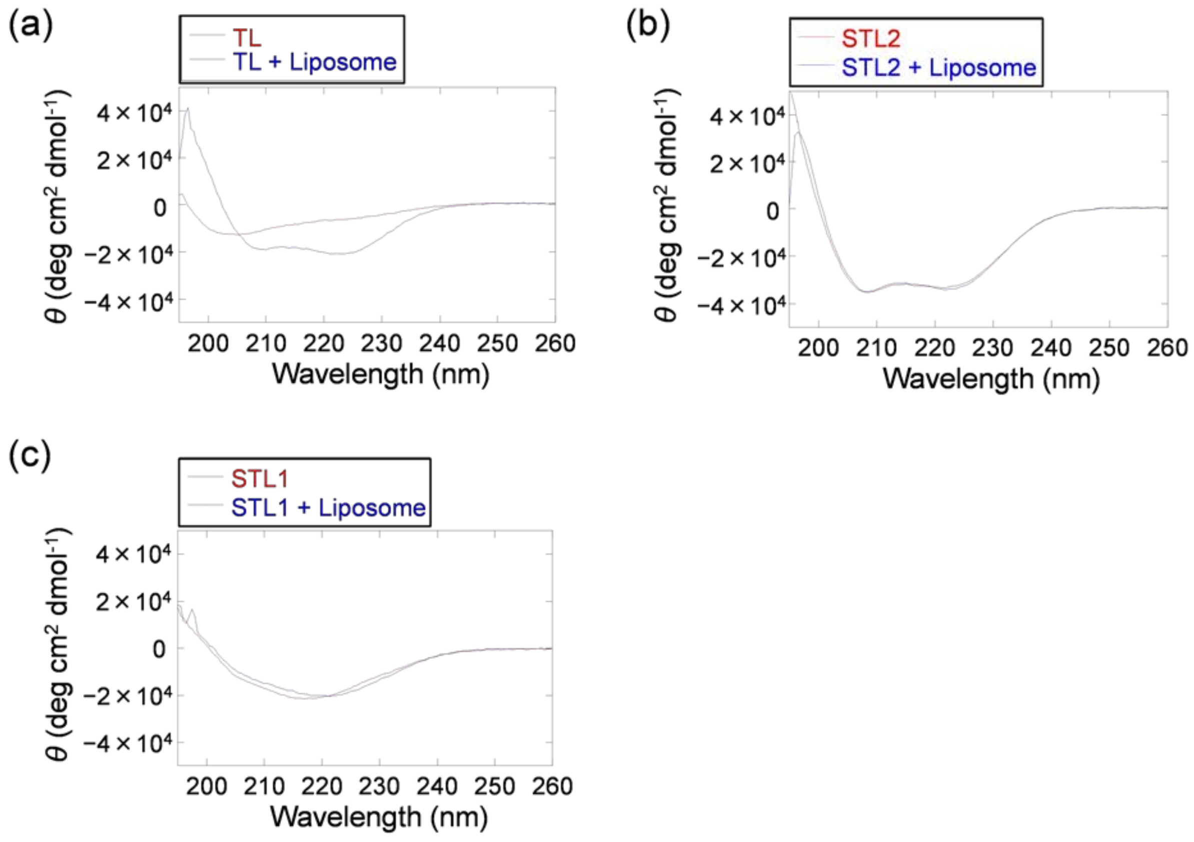

Figure S2. CD spectra of 50 μM TL (a), STL1 (b), and STL2 (c) with (blue line) or without (red line) liposome ([lipid] = 1.0 mg mL^{-1}) solution in 10 mM sodium phosphate buffer (pH = 7.4) containing 150 mM NaCl at 25 °C.

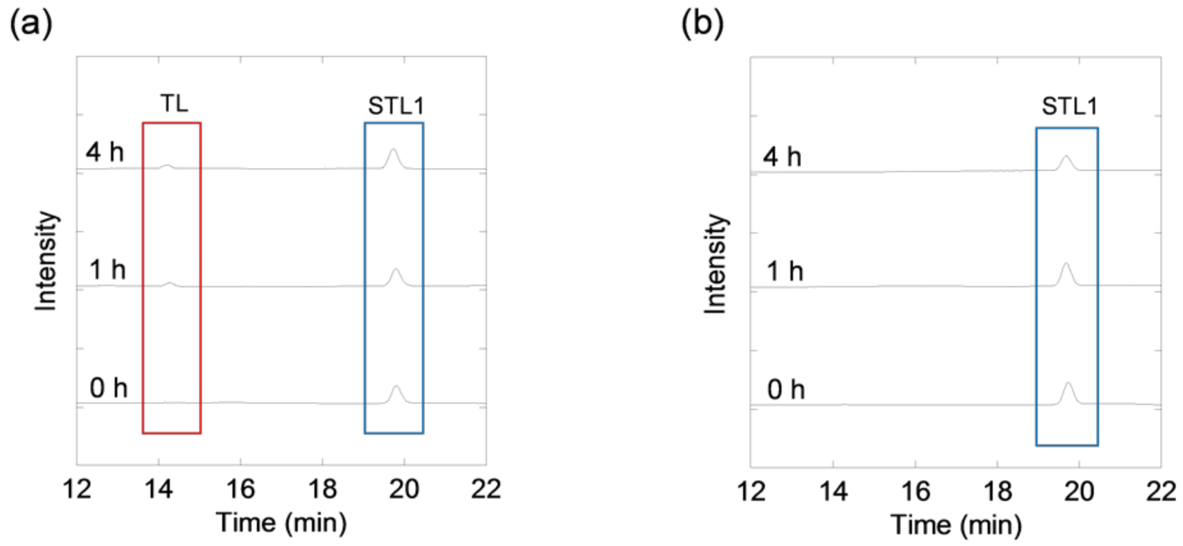

Figure S3. HPLC analysis of 25 μM STL1 with (a) and without (b) caspase-3 (1 U μL^{-1}). Eluent: 30%→55% CH_3CN/H_2O with 0.1% TFA, column: ODS, monitoring wavelength : 215 nm.

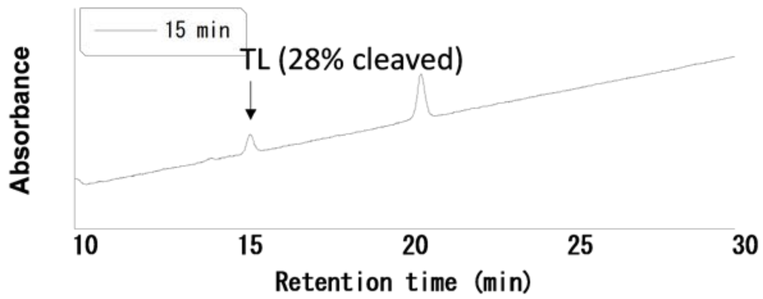

Figure S4. HPLC analysis of enzyme reaction. 100 μL of 3 μM STL1 was incubated with caspase-3 (1 U mL^{-1}) for 15min, Eluent: 30%→45%(30 min) CH_3CN/H_2O with 0.1% TFA

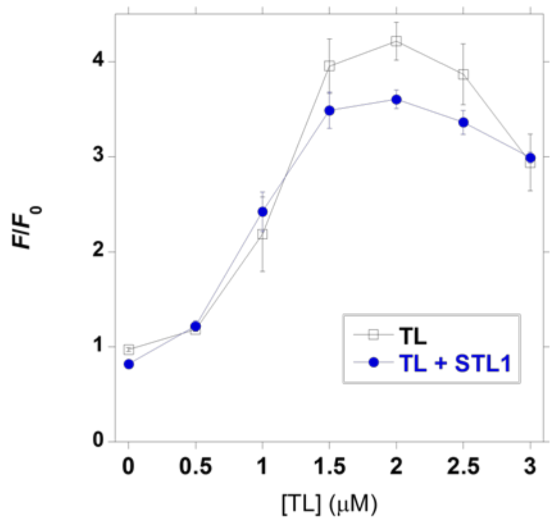

Figure S5. Membrane damaging activity of TL mixed (blue) with or (black) without STL1. Transverse axis refers to the actual concentration of TL. STL and TL were mixed to the total concentration = 3 μM. Each value was plotted as the mean ± S.D. (n = 3).

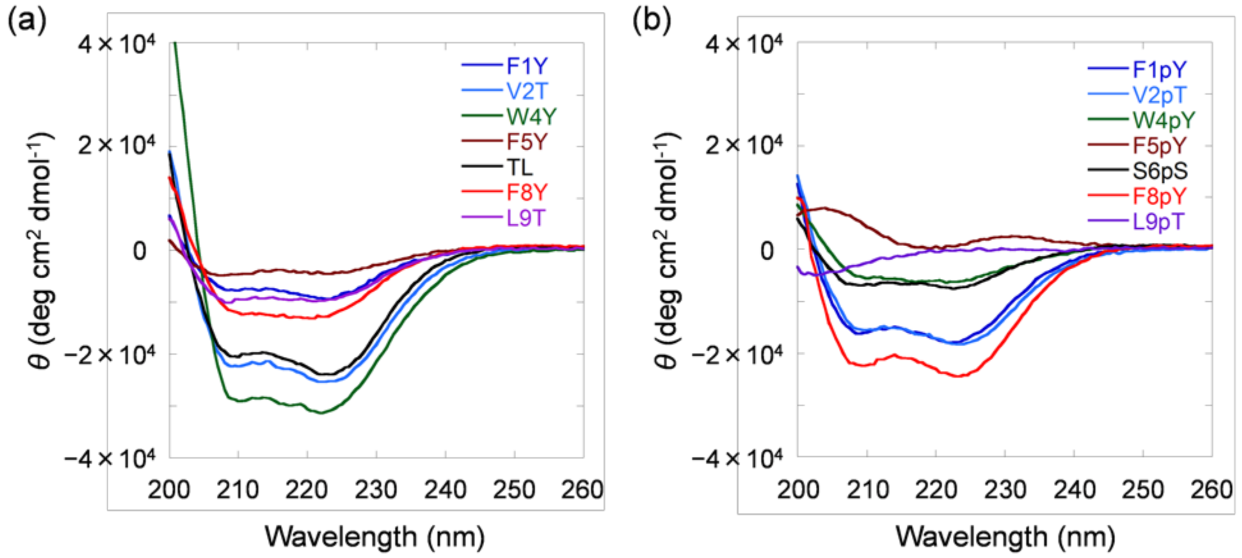

Figure S6. CD spectra of 50 μM non-phosphorylated (a) and phosphorylated (b) TL derivatives with liposome ([lipid] = 1.0 mg mL^{-1}) solution in 10 mM sodium phosphate buffer (pH = 7.4) containing 150 mM NaCl.

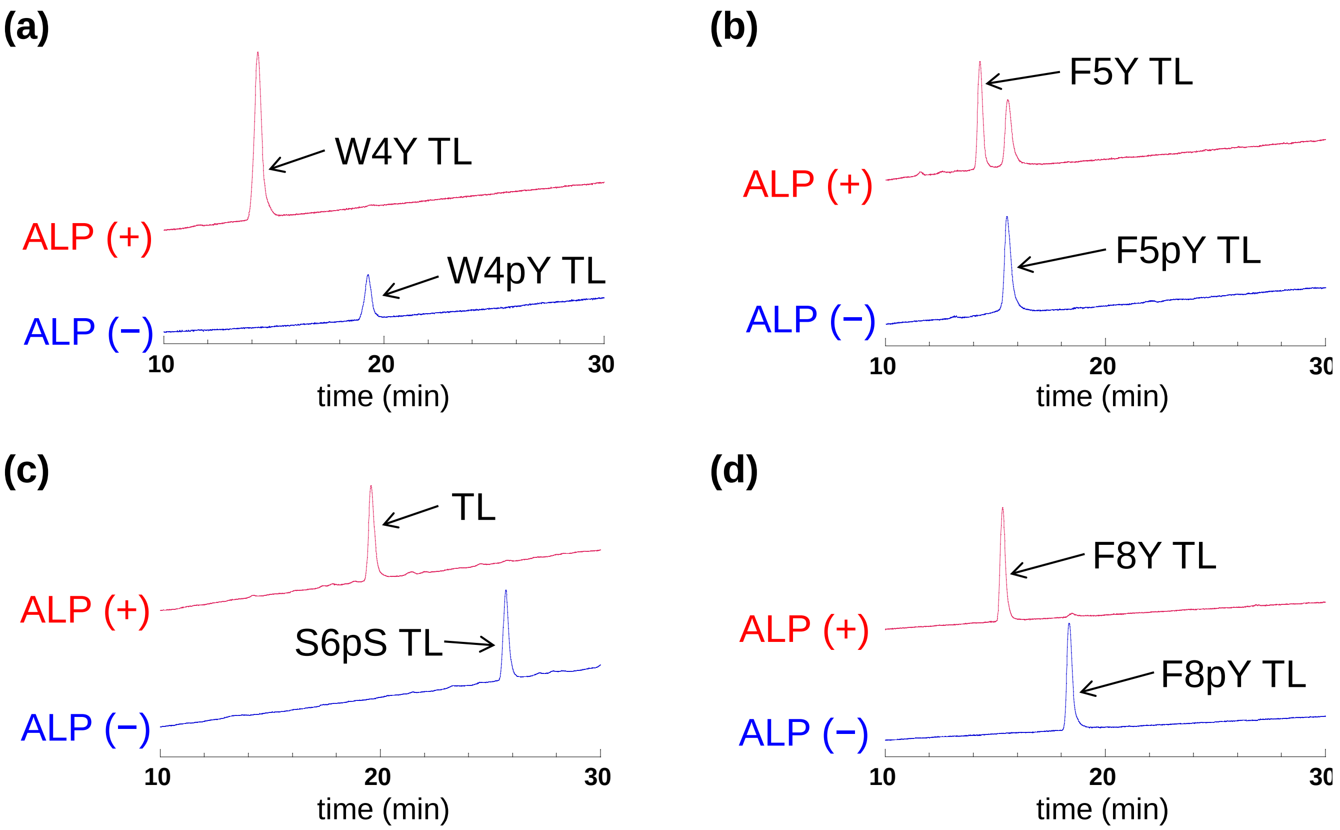

Figure S7. HPLC analyses of enzymatic dephosphorylation of phosphorylated TL derivatives by ALP. Reaction condition: 50 μM phosphorylated TL derivatives, 20 U mL^{-1} ALP in 10 mM HEPES buffer pH 7.4 containing 1 mM $MgCl_2$, and 150 mM NaCl at 37 ºC for 3 h. Eluent: 20%→35% CH_3CN/H_2O with 0.1% HCO_2H

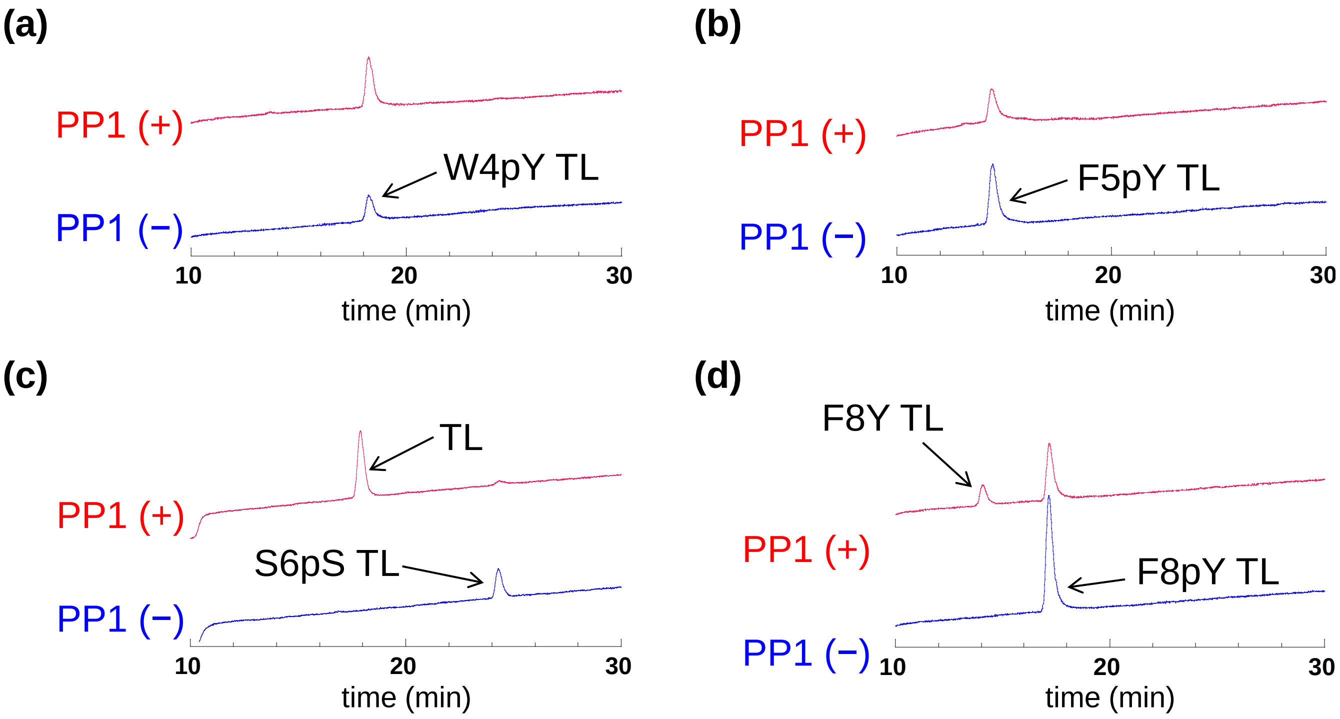

Figure S8. HPLC analyses of enzymatic dephosphorylation of phosphorylated TL derivatives by PP1. Reaction condition: 100 μM phosphorylated TL derivatives, 10 U mL^{-1} PP1 in 10 mM HEPES buffer (pH 7.4) containing 2 mM DTT, 1 mM $MnCl_2$, and 150 mM NaCl at 30 ˚C for 3 h. Eluent: 20%’ 35% CH_3CN/H_2O with 0.1% HCO_2H

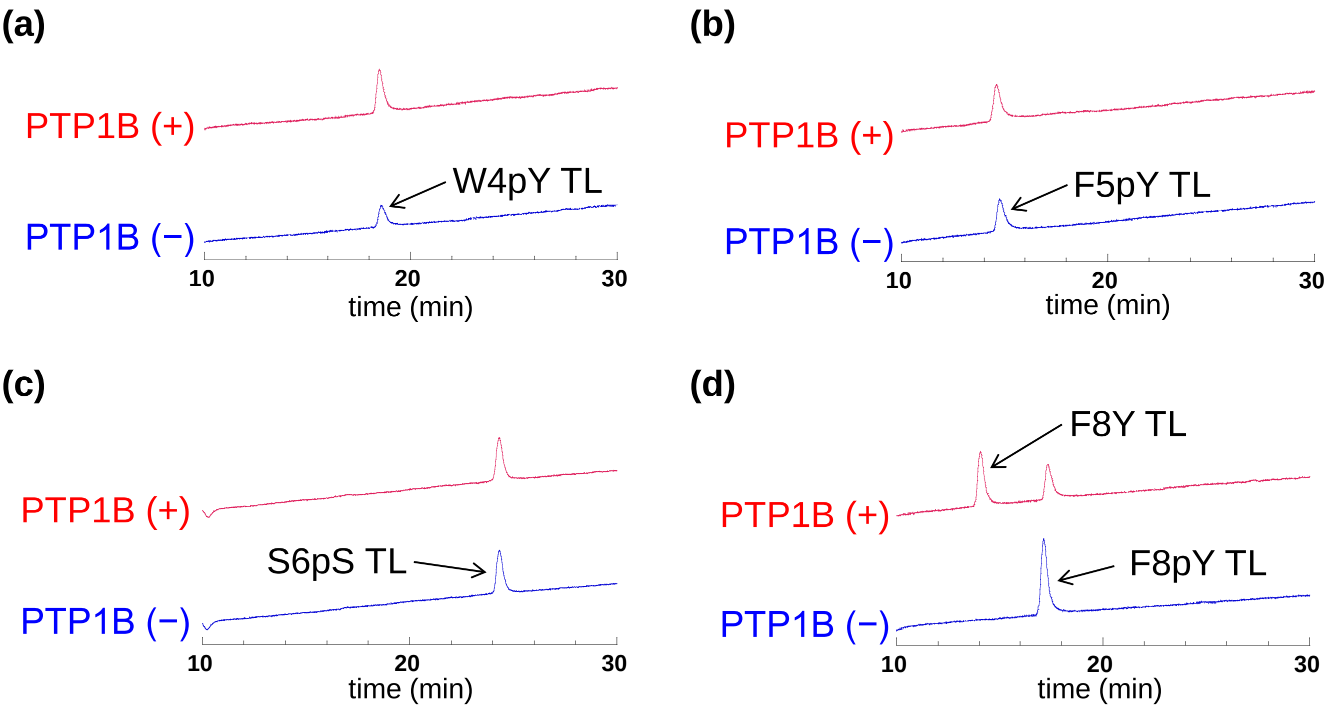

Figure S9. HPLC analyses of enzymatic dephosphorylation of phosphorylated TL derivatives by PTP1B. Reaction condition: 100 µM phosphorylated TL derivatives, 4 µg mL^{-1} (which corresponds to 24–48 U mL^{-1}) PTP1B in 10 mM HEPES buffer (pH 7.4) containing 5 mM DTT, 1 mM EDTA, and 150 mM NaCl at 37 ˚C. Eluent: 20%’ 35% CH_3CN/H_2O with 0.1% HCO_2H

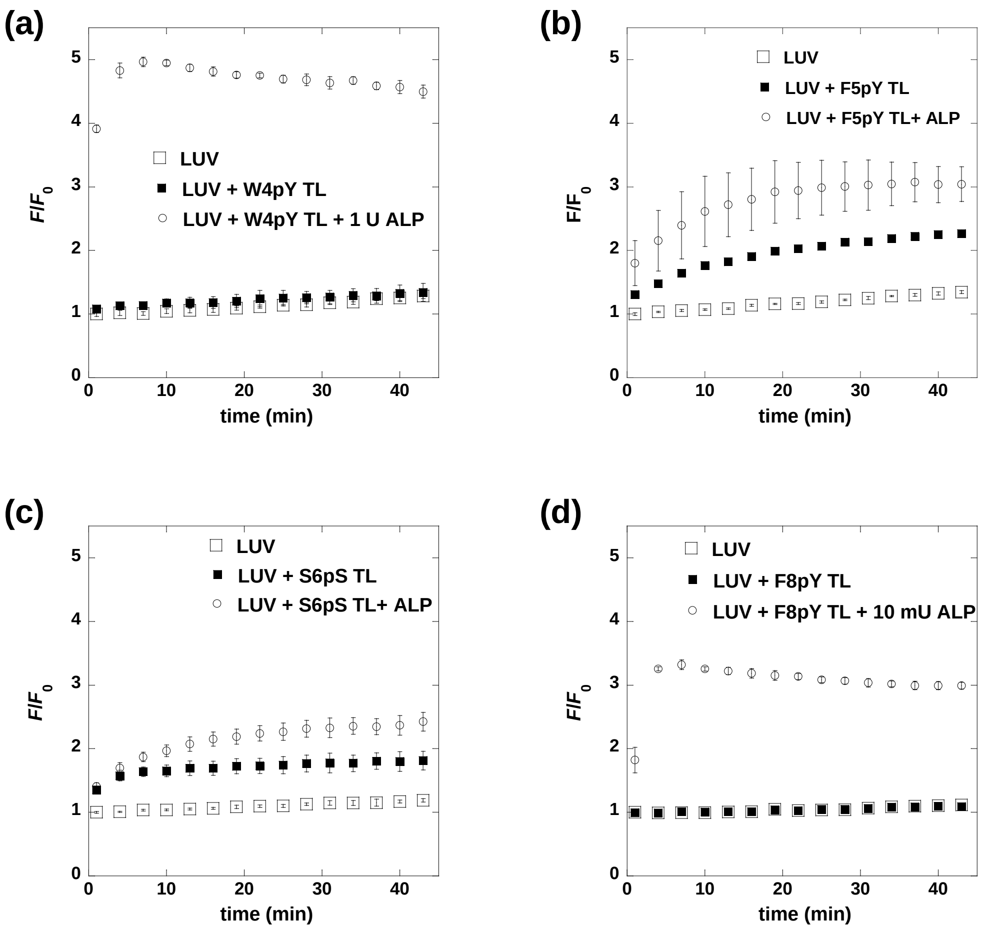

Figure S10. Real-time monitoring of ALP activity-triggered compound release with phosphorylated TL derivatives. [TL] = 3 µM, [ALP] = 10 U mL^{-1} (a–c) or 0.1 U mL^{-1} (d). Each value was plotted as the mean ± S.D. (n = 3).

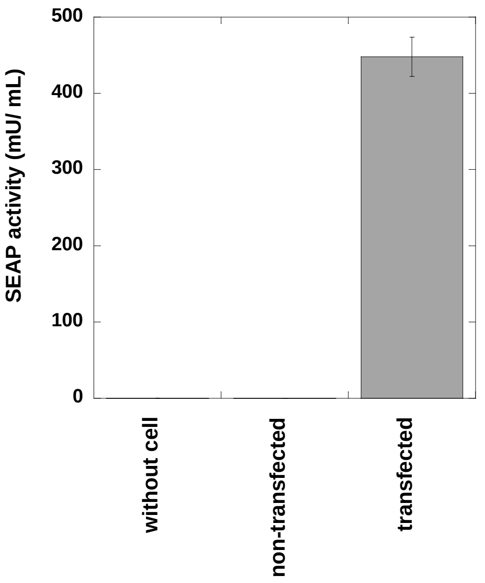

Figure S11. Quantification of SEAP activity from cultured medium of HEK 293 T cells. The cell culture medium was collected 48 h after the transfection, and SEAP activities were estimated by using external standard. About 450 mU mL^{-1} of SEAP was secreted from the transfected cells.

Table S1. Secondary structures and membrane damaging activities of synthesized TL derivatives.

Peptide	Structure[c]	Release (%)[b]	Peptide	Structure[c]	Release (%)[b]
**F1Y TL**	±-helix	93 ± 1	**F1pY TL**	±-helix	83 ± 1
**V2T TL**	±-helix	95 ± 7	**V2pT TL**	±-helix	37 ± 7
**W4Y TL**	±-helix	85 ± 3	**W4pY TL**	±-helix (weak)	−1.4 ± 1.0
**F5Y TL**	±-helix (weak)	77 ± 5	**F5pY TL**	N. D.[d]	1.3 ± 0.6
**TL**	±-helix	100 ± 1	**S6pS TL**	±-helix (weak)	2.7 ± 1.6
**F8Y TL**	±-helix	88 ± 5	**F8pY TL**	±-helix	3.0 ± 1.6
**L9T TL**	±-helix	1.4 ± 1.4	**L9pT TL**	N.D.	14 ± 1

[a] The different amino acid residue from original TL sequence was shown as a bold font. [b] All peptides were used at the concentration of 3 µM. Fluorescence increase by adding TL was normalized as 100%. [c] Secondary structures were predicted by the CD spectra at 50 µM with LUV ([lipid] = 1.0 mg mL^{-1}) (Figure S4). [d] Not determined.

Table S2. Dephosphorylated proportions of phosphopeptides in the presence of phosphatase.[a]

Enzyme	**W4pY TL**	**F5pY TL**	**S6pS TL**	**F8pY TL**
ALP	98	54	99	96
PP1	0	1	91	15
PTP1B	3	2	0	63

[a]Each value was determined by HPLC analysis (Figure S5–S7) after the incubation with each phosphatase and shown as percentage (%).

7. Supporting Reference

S1. J. Berger, J. Hauber, R. Hauber, R. Geiger and B. R. Cullen, *Gene*, **1988**, *66*, 1–10.

S2. I. Bronstein, J. Fortin, P. E. Stanley, G. S. Stewart and L. J. Kricka, *Anal Biochem*, **1994**, *219*, 169–181.

S3. Stewart, J. C. M. *Anal. Biochem.* **1980**, *104*, 10–14.
